# Negatively but Not Positively Charged Nanoceria Promoted Lateral Root Growth via Modulating the Distribution of Reactive Oxygen Species Rather than Auxin

**DOI:** 10.1002/gch2.202500186

**Published:** 2025-07-20

**Authors:** Guangjing Li, Jie Qi, Wenying Xu, Linlin Chen, Ashadu Nyande, Zhouli Xie, Jiangjiang Gu, Zhaohu Li, Honghong Wu

**Affiliations:** ^1^ National Key Laboratory for Germplasm Innovation & Utilization of Horticultural Crops, The Center of Crop Nanobiotechnology, College of Plant Science & Technology Huazhong Agricultural University Wuhan 430070 China; ^2^ Hubei Hongshan Laboratory Wuhan 430070 China; ^3^ College of Chemistry Huazhong Agricultural University Wuhan 430070 China; ^4^ Shenzhen Institute of Nutrition and Health Huazhong Agricultural University Shenzhen 511464 China; ^5^ Shenzhen Branch, Guangdong Laboratory for Lingnan Modern Agriculture, Genome Analysis Laboratory of the Ministry of Agriculture, Agricultural Genomics Institute at Shenzhen Chinese Academy of Agricultural Sciences Shenzhen 511464 China

**Keywords:** auxin, lateral root formation, nanoceria, ROS, transgenic lines

## Abstract

Lateral root (LR) formation is important for plant growth. ROS (reactive oxygen species)play an important role in LR formation. While how nanomaterials affect ROS distribution to promote LR formation and the role of ROS in primordia in LR formation are rarely known. Cerium oxide nanoparticles (nanoceria), as a potent ROS scavenger, are widely used in plants. This study investigates the effects of poly (acrylic acid) nanoceria (PNC, 6.5 nm, −36 mV), aminated nanoceria (ANC, 6.9 nm, 30 mV), and bulk nanoceria (BNC, 84.9 nm, −5.5 mV) on LR formation in *Arabidopsis*. Only PNC increased LR numbers by 73.5%, reducing root H_2_O_2_ levels by up to 90.44% and altering O₂^•−^ distribution in LR primordia (LRP). Furthermore, DPI (diphenyleneiodonium, O₂^•−^ inhibitor) decreased LR numbers by 18.9%, while PNC treatment reversed this inhibition (12.25 ± 0.53 vs 8.38 ± 0.52). Transcriptome analysis shows PNC regulated ROS metabolism via genes like peroxiredoxins and peroxidases, promoting LR formation. Interestingly, PNC does not affect auxin distribution (confirmed by DR5pro::GFP lines) or alleviate NPA‐induced (N‐1‐naphthylphthalamic acid, an auxin transport inhibitor) LR inhibition. These findings suggest that PNC enhances LR formation through ROS modulation rather than auxin signaling.

## Graphical abstract

PNC promote lateral root formation throughmodulating the dirstribution of superoxide anion rather than auxin.

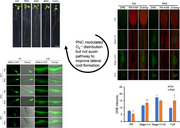

## 1 Introduction

Lateral roots (LRs) play a crucial role in plant fixation, plant water and nutrient absorption.^[^ ^1^ ^]^ The development of LRs enhances the absorptive capacity and surface area of the root system.^[^ ^2^ ^]^ Plants with larger LR systems always show better growth performance than those with smaller ones.^[^ ^3^ ^]^ Therefore, promoting LR formation in plants is of importance for efficient crop production and sustainable agriculture.^[^ ^4^ ^]^ Lateral root (LR) development is a complex process regulated by various substances, including hormones,^[^ ^5^ ^]^ signaling molecules, proteins, and organic compounds.^[^ ^6^ ^]^ Auxin^[^ ^7^ ^]^ and reactive oxygen species (ROS)^[^ ^8^ ^]^ play crucial roles in orchestrating this process. Auxin forms a gradient along the primary root, promoting pericycle cell division to initiate lateral root primordia (LRP).^[^ ^9^ ^]^ This gradient is maintained by auxin transporters, facilitating cell elongation and differentiation within the primordium.^[^ ^10^, ^11^, ^12^ ^]^ ROS also contributes to LR formation by increasing LR density and supporting auxin‐mediated cell wall remodeling in cortical cells. ROS accumulate in the apoplast of epidermal cells, where they are induced by respiratory burst oxidase homolog (RBOH).^[^ ^8^ ^]^ Together, auxin and ROS regulate key aspects of LR formation, including cell division, elongation, and vascular tissue patterning, ensuring proper root architecture.

Exogenous auxin treatments,^[^ ^13^ ^]^ genetic engineering,^[^ ^1^ ^]^ biochemical agents (such as auxin analogs),^[^ ^14^ ^]^ nutrient patches,^[^ ^15^ ^]^ and soil microbial interactions^[^ ^16^ ^]^ are established methods for stimulating lateral root (LR) formation. However, these approaches face challenges in practical applications due to issues such as environmental sensitivity,^[^ ^17^ ^]^ non‐specific physiological effects,^[^ ^18^ ^]^ high costs, and potential ecological risks.^[^ ^19^ ^]^ In contrast, nanobiotechnology, particularly the use of nanomaterials, offers a promising alternative due to its precision, stability, environmental compatibility, and sustainability. Nanomaterials have been shown to enhance plant growth,^[^ ^20^ ^]^ improve nutritional quality, and increase growth success rates.^[^ ^21^ ^]^ Despite the promising potential of nanomaterials, the mechanisms underlying their impact on LR formation remain poorly understood. Recent studies suggest that ROS and auxin are key signaling components in nanomaterial‐mediated root development. For example, bismuth vanadate nanoparticles promote primary root and LR growth in *Arabidopsis* by modulating ROS levels and hormone‐related gene expression.^[^ ^22^ ^]^ Graphene oxide has been shown to affect IAA content and regulate rice root growth. While cerium oxide nanoparticles (nanoceria) are widely known to enhance plant stress tolerance, their effect on LR formation remains inconclusive.^[^ ^23^, ^24^ ^]^ Previous studies have shown that nanoceria with a neutral charge and a particle size of 25 nm have little effect on root development and LR formation in plants.^[^ ^25^ ^]^ In contrast, our prior research demonstrated that negatively charged, smaller‐sized PNC can stimulate LR formation, alongside a reduction in ROS levels and Ca^2^⁺ concentrations in roots.^[^ ^26^ ^]^ However, the mechanisms underlying these effects remain unclear. Specifically, it is still uncertain how a decrease in ROS levels can promote LR formation and whether auxin plays a critical role in this process. Further investigation is needed to elucidate the interactions between nanomaterials, ROS, and in promoting LR formation, which could lead to more targeted and effective strategies for improving root growth.

In this work, we synthesized nanoceria with different size and charge and investigated its biological role on the promoting LR formation in *Arabidopsis thaliana*. By using laser confocal imaging technique, we investigated the distribution of PNC and the level and distribution of ROS and auxin in roots. We further confirmed the role of PNC in stimulating LR formation by using DPI. Transcriptomic analysis was conducted to investigate the possible key genes/pathways involved in PNC improved lateral root formation. The auxin distribution in *Arabidopsis* root treated with/without PNC was illustrated with *DR5pro::GFP* lines. Moreover, IAA inhibitor NPA was used to assess the role of auxin in PNC improvement of LR formation.

## 2 Results

### 2.1 PNC, ANC, and BNC Characterization

The crystalline phase of PNC was characterized by X‐ray diffraction (XRD). As shown in Figure S1 (Supporting Information), the diffraction pattern exhibits characteristic peaks at 28.7° (111), 33.2° (200), 47.7° (220), 56.5° (311), 69.6° (400), and 76.9° (331), which correspond exclusively to the cubic fluorite structure of CeO₂ (Figure S1, Supporting Information). XPS analysis provided complementary insights into the surface chemical composition and electronic states. For PNC, the elemental composition was dominated by oxygen (O1s: 35.09%) and carbon (C1s: 59.10%), with a minor cerium contribution (Ce3d: 1.46%) and detectable nitrogen (N1s: 4.35%). ANC was obtained by amino modification of PNC. The elemental composition of ANC revealed a significant increase in nitrogen content (N1s: 10.43% vs 4.35% for PNC) and a concurrent decrease in oxygen content (O1s: 26.31% vs 35.09%), leading to a substantial reduction in the O/N ratio (2.52 vs 8.07) (Figure S2A, Supporting Information). This dramatic rise in surface nitrogen is a direct fingerprint of the introduced amino (─NH₂ or ─NH─) functional groups. Deconvoluted XPS spectra showing the surface valence states (Ce^3+^, Ce^4+^) of PNC and ANC. The peaks at 906.9, 887.3, 882.2, 907.2, and 881.9 correspond to Ce^3+^, whereas the peaks at 904.1, 900.4, 885.5, 904.5, 900.2, and 886.1 indicate Ce^4+^ (Figure S2B,C, Supporting Information). Furthermore, the Ce3d spectrum of ANC (Figure S2C, Supporting Information) exhibited distinct changes compared to PNC. While Ce^4⁺^ was still present, a new component assigned to Ce^3⁺^ became prominent, evidenced by the fitted peaks at ≈907.2 and 887.3 eV (Figure S2C, Supporting Information). This shift in cerium oxidation state suggests a potential interaction or coordination between the introduced amino groups and the surface cobalt species, altering the local electronic environment.

**Figure 1:**
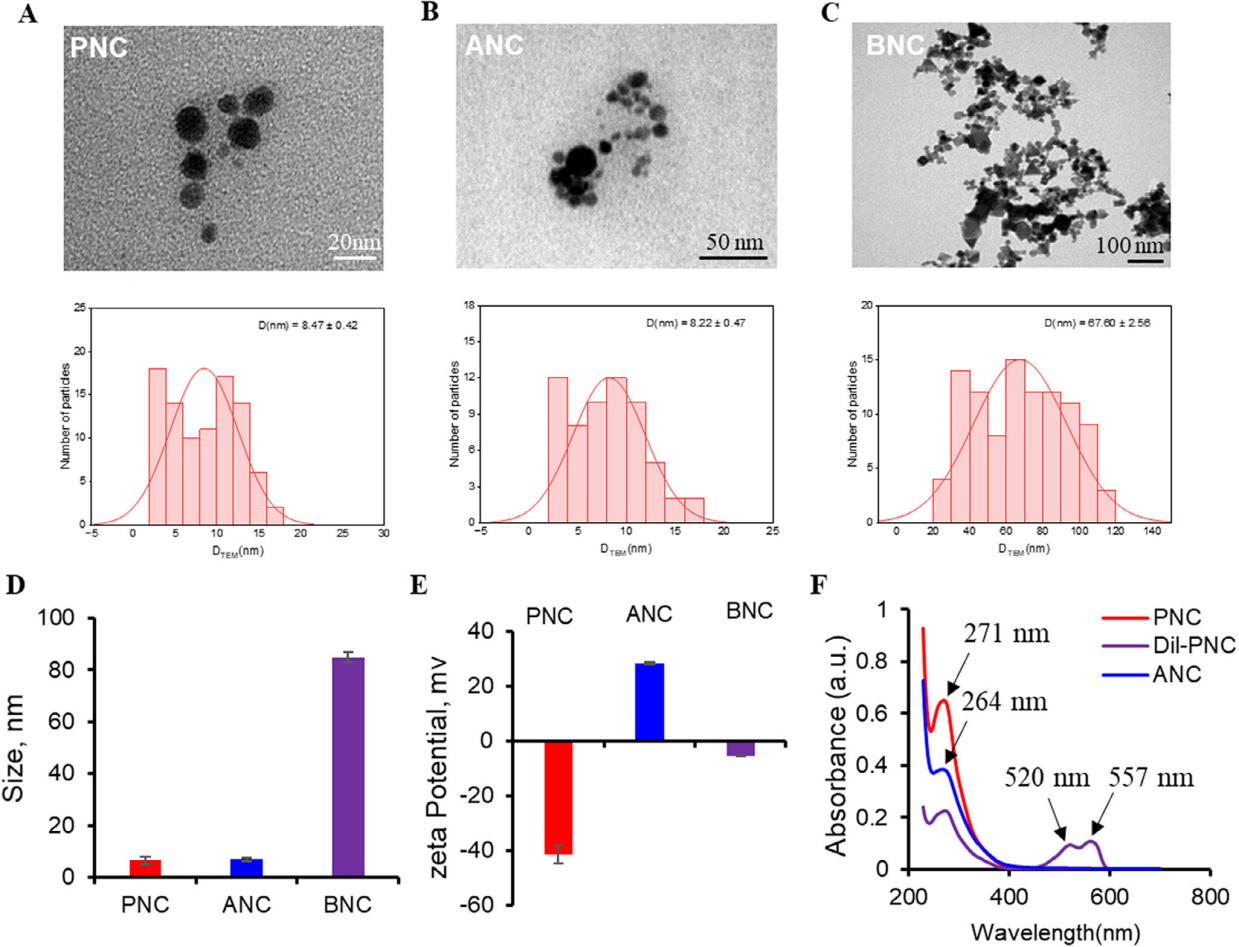
Characterization of cerium oxide nanoparticles. A–C) TEM image and particle size distribution calculated from TEM images of PNC (scale bar, 20 nm), ANC (scale bar, 50 nm), and BNC (scale bar, 100 nm). D,E) Measurement of hydrodynamic size (D) and ζ‐potential (E) for PNC, ANC, and BNC. F) UV–vis absorbance spectra of PNC, DiI‐PNC, and ANC. Mean ± SE (n = 3–6). At least three independent batches were tested.

The PNC, ANC and BNC were characterized by transmission electron microscopy (TEM). ANC are derived from PNC, sharing the same core. TEM images showed that PNC and ANC were well dispersed (**Figure** **1**A,B), while BNC were with irregular shape showing the mixing of diamond, rectangle, and spherical shapes (Figure 1C). The hydrodynamic diameters of PNC and ANC, measured by using dynamic light scattering, were 6.5 ± 1.7 nm and 6.9 ± 0.6 nm, respectively, which is similar with previous study.^[^ ^27^ ^]^ BNC exhibits a larger hydrodynamic diameter of 84.9 ± 1.9 nm (Figure 1D; Figure S3, Supporting Information). ζ‐potential results showed that PNC (−41.5 ± 3.0 mV) and ANC (28.3 ± 0.6 mV) are charged, compared with the neutral charge of BNC (−5.5 ± 0.3 mV) (Figure 1E). FTIR spectroscopy was employed to further characterize the surface functional groups of the nanoparticles (Figure S4, Supporting Information). The spectrum of the PNC exhibited characteristic peaks of polyacrylic acid (PAA), confirming the presence of surface ─COOH. No significant changes of FTIR spectra around 1200–1800 cm^−1^ were observed between PNC and DiI‐PNC. It is known that DiI dyes do not coat the surface of nanoceria, while it can encapsulate inside the hydrophobic polymer shell of PNC.^[^ ^27^, ^32^ ^]^ Due to the amino functionalization on ANC, the attenuation of the carboxylic acid C═O stretch and the emergence of distinct ─NH were observed. This provides direct evidence for the conversion of surface carboxyl groups to amide linkages. In contrast, the spectrum of BNC displayed minimal features in the organic functional group region, consistent with its identity as unmodified CeO₂. The UV–vis spectra showed that PNC and ANC had characteristic absorption peaks at 271 and 264 nm, respectively. To track the root distribution of nanoparticles, PNC and ANC were labelled with the fluorescent dye 1,1′‐dioctadecyl‐3,3,3′,3‐tetramethylindocarbocyanine perchlorate (DiI). DiI‐PNC showed absorption peaks at 271, 520, and 557 nm, indicating successful coating of the DiI to the PNC (Figure 1F).

### 2.2 Negatively Charged PNC Increased the Number of LRs

**Figure 2:**
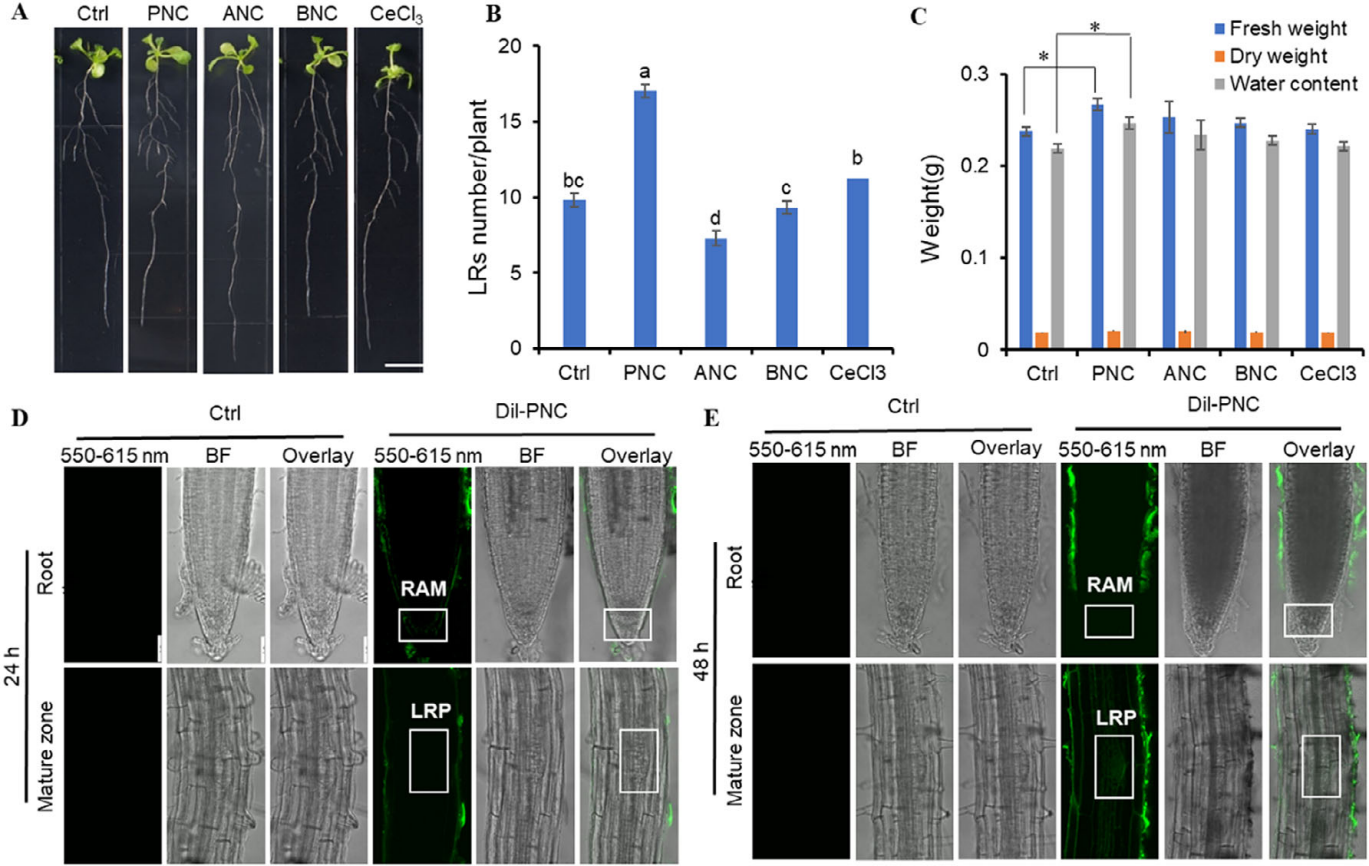
*Arabidopsis* lateral root formation and distribution of cerium oxide nanoparticles (PNC) in roots. A) Phenotype of *Arabidopsis* seedlings treated with 0.1 mM various cerium oxide nanoparticles for 7 days. Scale bar, 1 cm. B) Number of lateral roots per plant in *Arabidopsis* seedlings after 7 days of treatment. Mean ± SE (n = 3 biological replicates. Each biological replicates contains eight plants). The comparison was performed by one‐way ANOVA based on Duncan's multiple range test. Different lowercase letters mean the significance at *p* < 0.05. C) Fresh and dry weight of roots of *Arabidopsis* after 7 days of treatment. Mean ± SE (n = 3 biological replicates. Each biological replicates contains eight plants). The comparison was performed by an independent sample t‐test. * *p* < 0.05, ** *p* < 0.01. D,E) Confocal images showing the distribution of PNC in *Arabidopsis* roots at D) 24 h and E) 48 h post‐treatment. White box indicates root apical meristem (RAM) and lateral root primordia (LRP), respectively. Scale bar, 50 µm. Mean ± SE (n = 6 biological replicates. Each biological replicate contains three plants).

Compared with the control treatment, the application of 0.1 mm PNC (smaller particle size) led to a significant increase in the number of LRs per plant (17.04 ± 0.43 vs 9.83 ± 0.46, **Figure** **2**A,B), along with a 12.4% increase in root fresh weight (0.23 ± 0.007 vs 0.21 ± 0.004 g) and a 9.5% increase in dry weight (18 ± 0.01 vs 16 ± 0.07 mg, Figure 2C). In contrast, BNC (larger particle size) showed no significant difference in LR numbers compared to the control (9.32 ± 0.42 vs 9.83 ± 0.46; Figure 2B), indicating nanoparticle size as a critical factor influencing LR formation. Notably, ANC induced an effect diametrically opposed to that of PNC on LRs formation in *Arabidopsis*, resulting in a significant reduction of 25.7% in the number of LRs (7.30 ± 0.47 vs 9.83 ± 0.46, Figure 2A,B). CeCl_3_ treatment did not alter LR numbers (11.27 ± 0.40 vs 9.83 ± 0.46; Figure 2A,B), suggesting nanoceria‐induced LR promotion is unrelated to cerium ions. Furthermore, our results showed PNC could also improve LR formation in rapeseed and rice (Figure S5, Supporting Information), showing the broad application potential of applying PNC to promote LR formation. Confocal imaging results showed a predominant accumulation of DiI‐PNC in the root apical meristem (RAM) area after 24 h treatment (Figure 2D). After 48 h treatment, in comparison to 24 h, we observed intensified fluorescence signals on the outer epidermis of the root tip, with fluorescence accumulation notably in cells within the mature zone, particularly in the LRP (Figure 2E). Compared with DiI‐PNC, the fluorescence signal of DiI‐ANC remained confined to the epidermis at both 24 and 48 h post‐treatment, failing to penetrate the RAM or LRP (Figure S6, Supporting Information). This observation aligns with prior studies indicating that the altered charge of ANC facilitates its adsorption by root exudates, thereby restricting internalization into root tissues.^[^ ^33^, ^34^ ^]^

### 2.3 PNC Modulate ROS Level in *Arabidopsis* Root Tip and LR Primordia

**Figure 3:**
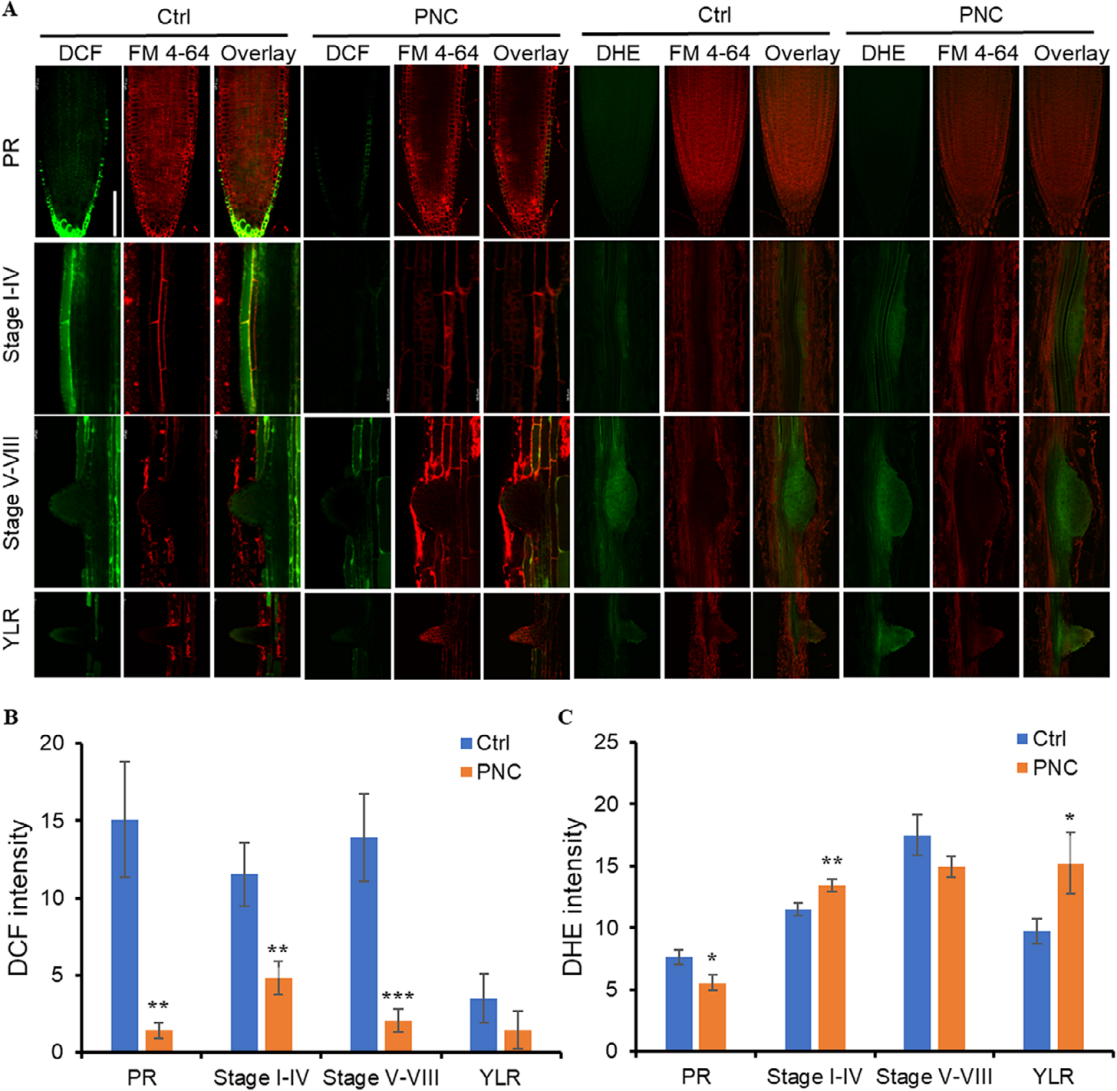
ROS content in *Arabidopsis* roots treated with PNC for 7 days. A) Confocal images showing the fluorescence of DCF and DHE in *Arabidopsis* roots after PNC treatment for 7 days. Scale bar, 100 µm. B,C) Calculated intensity of B) DCF and C) DHE in different developmental stages of LRP. YLR: young lateral root. Mean ± SE (n = 8–10 biological replicates). The comparison was performed by an independent sample t‐test. * *p* < 0.05, ** *p* < 0.01, *** *p* < 0.001.

Confocal imaging using DCFH‐DA (H_2_O_2_ indicator), DHE (O₂^•−^ indicator), and FM 4–64 (membrane stain) revealed altered ROS dynamics in PNC‐treated *Arabidopsis* roots. PNC significantly reduced H_2_O_2_ levels (DCF fluorescence) in root tips (1.44 ± 0.50 vs 15.09 ± 3.72; 90.4% decrease), young LRs (1.47 ± 1.25 vs 3.50 ± 1.56; 57.9% decrease), and LRP at stages I‐IV (4.80 ± 1.08 vs 11.53 ± 2.07; 58.3% decrease) and stages V‐VIII (2.05 ± 0.73 vs 13.92 ± 2.82; 85.3% decrease) compared to control group (**Figure** **3**A,B). PNC treatment also modulated O₂^•−^ levels (DHE fluorescence), with a 27.2% reduction in root tips (5.55 ± 0.65 vs 7.62 ± 0.63) but a 16.7% increase in early‐stage LRP (I‐IV: 13.42 ± 0.53 vs 11.50 ± 0.50) and a 57.9% rise in young LRs (15.2 ± 2.48 vs 9.69 ± 1.01). No significant differences were observed in late‐stage LRP (V‐VIII: 14.92 ± 0.83 vs 17.47 ± 1.65) (Figure 3A,C). Not surprisingly, O₂^•−^ inhibitor DPI (1 µM, diphenylene iodonium) decreased the LR numbers per plant by 18.9% (8.38 ± 0.52 vs 10.33 ± 0.5, Figure S7A,B, Supporting Information). While, co‐application of PNC and DPI completely alleviated the inhibitory effect observed with DPI treatment alone on LR numbers in *Arabidopsis* (12.25 ± 0.53 vs 8.38 ± 0.52, Figure S7A,B, Supporting Information). Furthermore, this combined treatment exhibited a root‐promoting effect comparable to that of PNC alone (12.25 ± 0.53 vs 13.17 ± 0.54, Figure S7A,B, Supporting Information).

### 2.4 PNC Alter ROS Distribution via Regulation of Metabolism‐Related Genes

**Figure 4:**
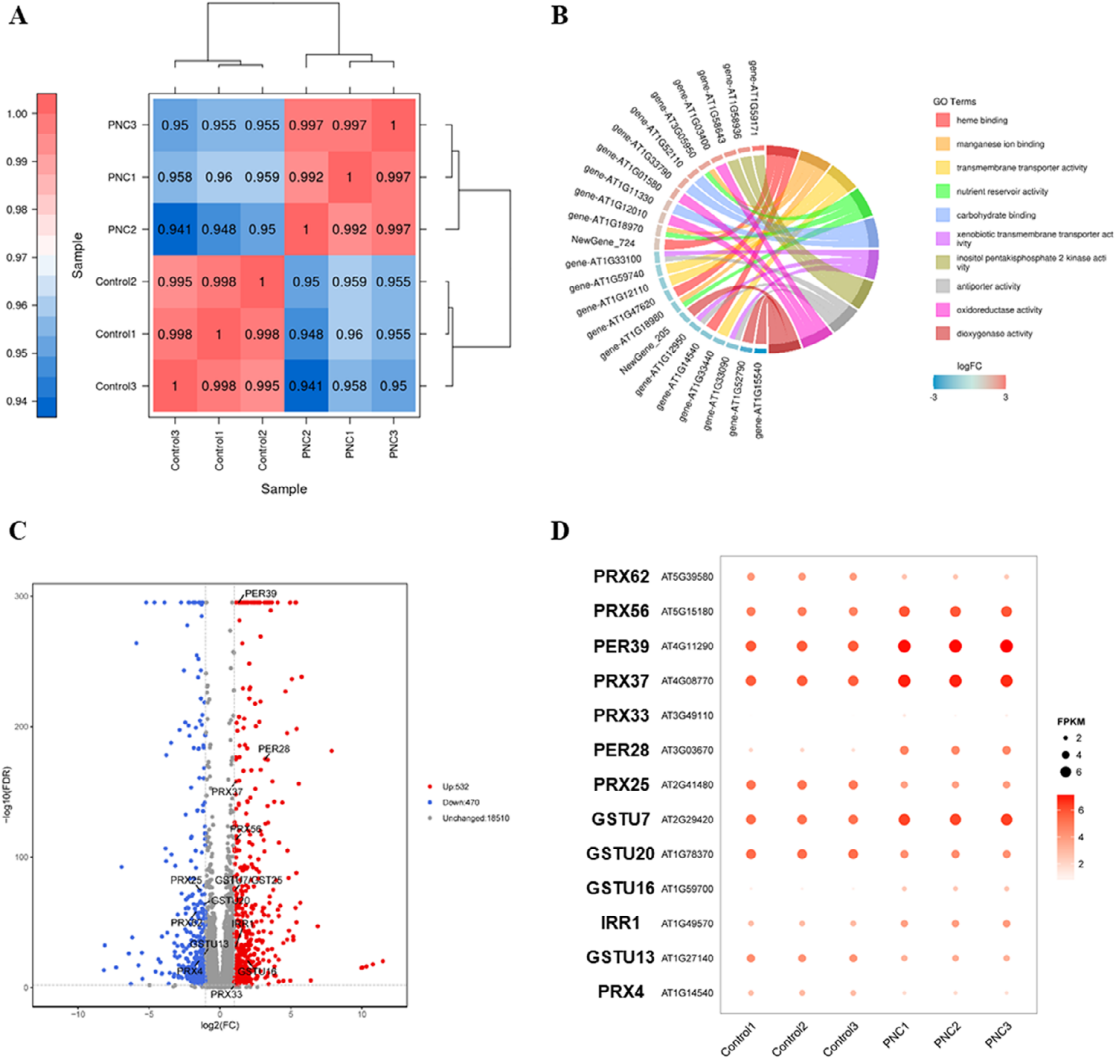
Comprehensive analysis of differential gene expression and functional enrichment in *Arabidopsis* roots under PNC treatment. A) Correlation heatmap of samples. B) Chord diagram of GO enrichment analysis for DEGs in *Arabidopsis* roots under PNC treatments. C) Volcano plot of DEGs between control and PNC treatment. Red dots represent upregulated genes, blue dots represent downregulated genes (FC ≥ 2; FDR < 0.01). D) Differential expression of oxidoreductase genes in *Arabidopsis* roots comparing control and PNC treatment groups. Mean ± SE (n = 3 biological replicates. Each replicate contains four plants).

Transcriptome datasets for control and PNC‐treated samples were highly correlated (Pearson coefficient > 0.9), indicating good reproducibility across biological replicates (**Figure** **4**A). Gene Ontology (GO) enrichment analysis revealed significant associations between differentially expressed genes (DEGs) and oxidoreductase and membrane transporter activities, suggesting these biological processes in *Arabidopsis* root are primarily affected by PNC treatment (Figure 4B). A total of 1002 DEGs were identified (FDR < 0.05, fold change ≥ 2), with 532 upregulated genes and 470 downregulated genes (Figure 4C). Notably, genes associated with ROS metabolism showed pronounced expression changes. Peroxiredoxins (PRX56, PRX37) and peroxidases (PER28, PER39) were significantly upregulated, while PRX62, PRX25, and PRX4 were downregulate Glutathione transferase genes (GSTU7, GST16) were also upregulated, whereas GST20 and GST13 exhibited reduced expression (Figure 4D). These findings suggest a coordinated modulation of ROS homeostasis in PNC‐treated roots.

### 2.5 PNC Promoted LR Formation Bypassed the Auxin Pathway

**Figure 5:**
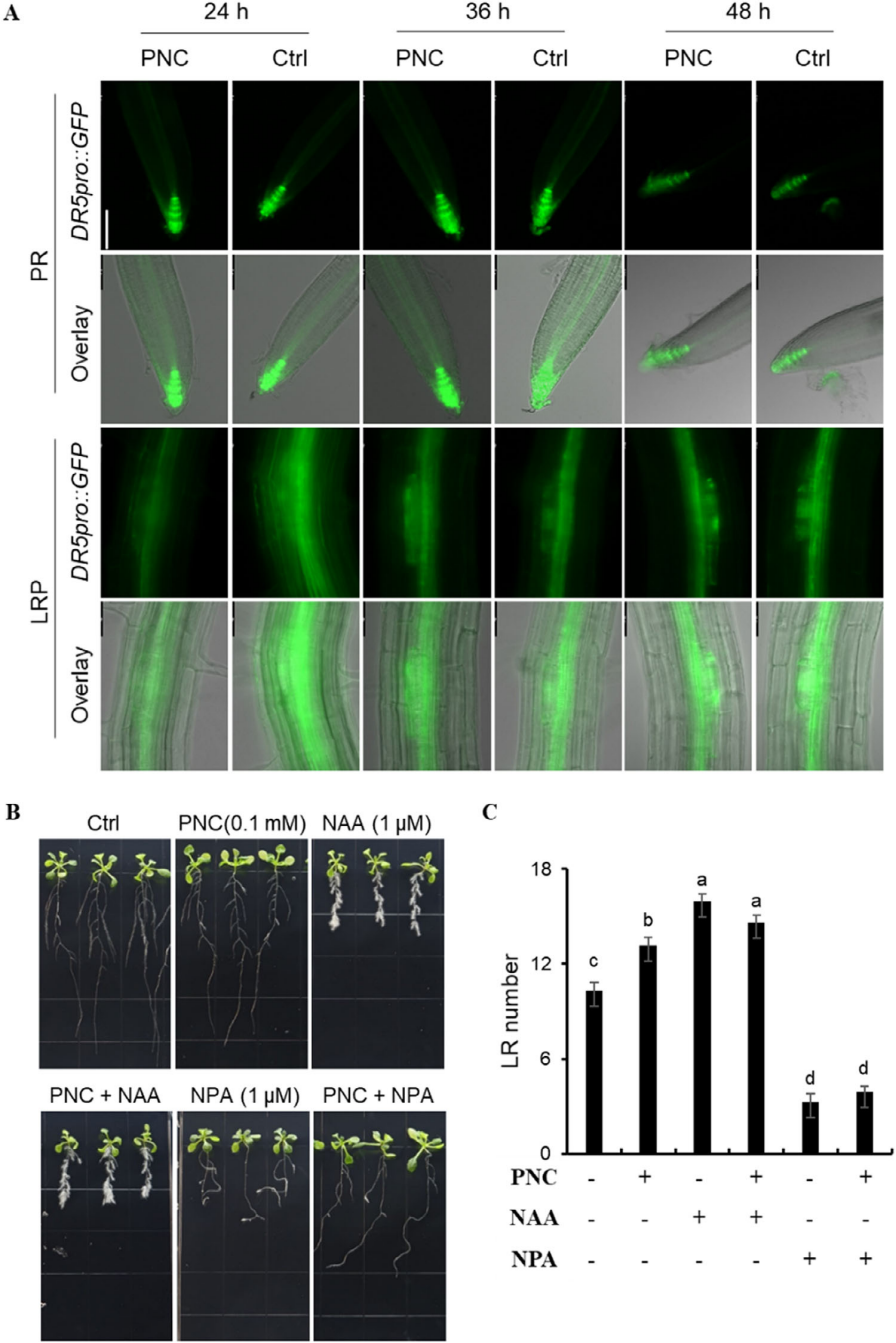
Effect of PNC treatment on auxin distribution and lateral root formation in *Arabidopsis*. A) GFP fluorescence imaging of DR5pro::GFP *Arabidopsis* roots exposed to PNC for 24, 36, and 48 h, showing consistent auxin distribution patterns across all time points. PR: primary root, LRP: lateral root primordium. Scale bar, 100 µm. 12 plants were used. B) Phenotypes of *Arabidopsis* seedlings treated with PNC, NAA, and NPA for 7 days. Scale bar, 1 cm. C) Number of lateral roots in *Arabidopsis* after 7 days of treatment with PNC, NAA, and NPA. Mean ± SE (n = 6 biological replicates, each biological replicate contains three plants). The comparison was performed by one‐way ANOVA based on Duncan's multiple range test. Different lowercase letters mean the significance at *p* < 0.05.

Auxin plays a crucial role in regulating various aspects of plant growth and development.^[^ ^35^ ^]^ To assess the effect of PNC treatment on auxin distribution in *Arabidopsis* roots, we utilized DR5 transgenic *Arabidopsis* seedlings equipped with GFP fluorescent markers to visualize auxin distribution at 24, 36, and 48 h after PNC application (**Figure** **5**A). Interestingly, no significant changes in auxin distribution were detected following 24, 36, or 48 h of PNC treatment when compared to the control group (Figure 5A). This is different with the results in previous studies showing that auxin plays an important role in promoting LR numbers in plants.^[^ ^36^ ^]^ To explore whether PNC promotes LR formation in *Arabidopsis* through the auxin pathway, *Arabidopsis* seedlings were treated in vitro with the synthetic auxin NAA and the auxin polar transport inhibitor NPA. NAA treatment alone led to a significant increase of 54.3% in LR numbers in *Arabidopsis* compared to the control group (15.94 ± 0.48 vs 10.33 ± 0.50, Figure 5B,C). However, co‐application of NAA and PNC did not show higher LR numbers than NAA treatment alone (14.63 ± 0.45 vs 15.94 ± 0.48, Figure 5C). NPA inhibits the polar transport of auxin, leading to a decreased ability of the *Arabidopsis* roots to grow vertically, and subsequently, a reduction in LR formation.^[^ ^36^ ^]^ Following NPA treatment, the number of LRs in *Arabidopsis* decreased markedly by 31.8% of the control group (3.29 ± 0.49 vs 10.33 ± 0.50, Figure 5C). In the presence of NPA, the addition of PNC can partially restore the degree of root curvature of *Arabidopsis*, but it does not significantly increase the number of LRs 3.92 ± 0.32 vs 3.29 ± 0.49) (Figure 5B,C).

## 3 Discussion

### 3.1 Negatively Charged Nanomaterials are more Pronounced in Promoting LR Formation

The surface charge and size of nanomaterials are critical determinants of their interactions with plant roots, influencing uptake, mobility, and subsequent physiological effects.^[^ ^37^, ^38^, ^39^ ^]^ Our findings demonstrate that negatively charged nanoceria (PNC, 6.5 nm) significantly promoted LR formation in *Arabidopsis*, resulting in a 73.5% increase relative to the control, whereas their positively charged nanoceria (ANC) suppressed LR development (Figure 2B). This aligns with previous studies showing that anionic nanoparticles exhibit superior biocompatibility and uptake efficiency in plants. For example, compared with positively charged carbon dots (+43 mV), negatively charged carbon dots (−38 mV) were more efficiently translocated from root to shoot via employing symplastic and apoplastic pathways.^[^ ^40^ ^]^ Similarly, anionic gold nanoparticles accumulate in root meristems, as visualized by hyperspectral imaging,^[^ ^34^ ^]^ underscoring a universal preference for negatively charged nanomaterials in plant systems. Conversely, cationic or larger nanoparticles have been usually associated with phytotoxicity or restricted mobility within plant tissues.^[^ ^41^ ^]^

It is known that the plant cell wall, a dynamic and charge‐selective barrier, plays a pivotal role in nanoparticle uptake. Its porosity (≈13 nm) physically restricts larger particles. Nanoparticles with big sizes often exhibit phytotoxicity or limited mobility.^[^ ^42^, ^43^ ^]^ Smaller nanoparticles like PNC in this study (≈10 nm) bypass this size exclusion, while their negatively charged surface enhances their internalization by binding to the cell wall.^[^ ^44^ ^]^ This dual filtering mechanism—size sieving and charge affinity—explains the preferential accumulation of PNC in LRP (Figure 2D), where thin cell walls and high metabolic activity further enhance the delivery efficiency of nanoparticles. Once internalized, these nanoparticles may alter the root microenvironment—potentially through modulation of ion gradients, redox states, or cell wall remodeling enzymes—thereby triggering the asymmetric division of pericycle cells, a pivotal event in LR initiation.^[^ ^45^, ^46^ ^]^ This hypothesis is corroborated by the observed colocalization of PNC with LRP 48 h (Figure 2E). Collectively, these findings highlight the potential of anionic nanomaterials as tools for precision LR engineering, offering broader implications for optimizing plant growth and resilience in agricultural applications.

### 3.2 The Distribution of Superoxide Anion in Primordia is Critical for Nanoceria Promoted LR Formation

ROS exhibit spatiotemporal specificity in regulating LR development, with distinct roles for H₂O₂ and O₂^•−^. While exogenous H₂O₂ promotes LR emergence through cortical cell wall remodeling,^[^ ^8^ ^]^ our study reveals that PNC enhances LR formation via an O₂^•−^‐driven mechanism. Confocal imaging results demonstrated a 16.7% increase in O₂^•−^ fluorescence intensity specifically within the early‐stage LRP (stages I–IV) under PNC treatment (Figure 3C). This spatially restricted O₂^•−^ accumulation contrasts with the uniform H₂O₂ distribution observed in control roots, suggesting a divergence from typical ROS‐mediated pathways. O₂^•−^ exerts multifaceted effects on plant growth and development. Previous studies demonstrated that the concentration gradient of O₂^•−^ plays a critical role in modulating cell differentiation.^[^ ^47^, ^48^ ^]^ During xylem development, O₂^•−^ directly contributes to cell wall expansion and remodeling.^[^ ^49^ ^]^ In this study, the functional necessity of O₂^•−^ spatial patterning was validated by pharmacological inhibition: treatment with diphenyleneiodonium (DPI), an O₂^•−^ inhibitor, abolished LR initiation by depleting O₂^•−^ in LRP meristems (Figure S7A,B). Importantly, PNC not only restored O₂^•−^ levels in these regions but also fully rescued LR formation (12.25 ± 0.53 vs 8.38 ± 0.52), directly linking O₂^•−^ localization—not merely its abundance—to developmental outcomes. This contrasts with studies in cucumber, where PNC elevated H₂O₂ levels in root apices,^[^ ^50^ ^]^ highlighting species‐specific ROS modulation by nanomaterials, potentially influenced by root anatomy, exudate composition, or antioxidant capacity.

It is known that the spatial regulation of ROS signals by distinct RBOH isoforms is important for controlling LR formation. For example, H_2_O_2_ and O₂^•−^ are known to promote LR formation.^[^ ^8^, ^51^ ^]^ However, it should be noted that H₂O₂ derived from RBOHD/F inhibits LR development.^[^ ^51^, ^52^ ^]^ These results suggest the complex role of RBOHs in controlling LR formation. PNC are known as a potent ROS scavenger which have good ability to scavenge H_2_O_2_ and O₂^•^O₂^•−^. Herein, our results showed that compared with control plants, PNC treated plants showed significantly lower H_2_O_2_ content in primary root and lateral roots (stage I‐IV and stage V‐VIII), while only young lateral roots and lateral roots (stage I‐IV) had significant difference (increase) regarding O₂^•−^ content. Also, the inhibition of lateral root formation by the RBOH inhibitor DPI, along with the rescue effect of PNC on DPI treatment, further confirming the important role of O₂^•−^ in LR formation. Together, these results suggest that compared with H_2_O_2_, O₂^•−^ played a more important role in promoting LR formation, at least in *Arabidopsis*. RBOH is known as a source of H_2_O_2_ and O₂^•−^. Thus, the fine tune of RBOH role in modulating LR formation should be further investigated in future studies. Here, we propose that PNC may create a micro‐domain of O₂^•−^ by selectively activating RBOH subtypes, such as RBOHE,^[^ ^8^ ^]^ that promote lateral root development. The specific accumulation of O₂^•−^ in the LRP meristem demonstrates significant co‐localization with the spatial expression pattern of RBOHE in the surrounding tissue.^[^ ^8^ ^]^ Notably, diffuse peroxidase‐derived O₂^•−^ present in the rbohd/f mutant could not rescue the developmental defects caused by localized RBOH deficiency.^[^ ^51^ ^]^ Overall, we propose that micro‐domain of O₂^•−^ which is associated with the fine tune of RBOHs may function as a spatiotemporal regulatory molecule, precisely triggering stage‐specific processes to promote LR formation.

Transcriptomic analysis revealed that PNC treatment significantly upregulated genes encoding peroxiredoxins (*PRX37*, *PRX56*) and glutathione transferases (*GSTU7*, *GST16*) (Figure 4D). PRX is known to scavenge excess H₂O₂, mitigating oxidative stress,^[^ ^53^ ^]^ while GSTU maintains glutathione homeostasis, essential for redox buffering.^[^ ^54^, ^55^ ^]^ Together, these enzymes create a redox microenvironment that sustains localized O₂^•−^ accumulation in LRP meristems. This localized O₂^•−^ burst is critical for activating NADPH oxidases (RBOHD/F), which further amplify O₂^•−^ production through a self‐reinforcing loop,^[^ ^56^ ^]^ thereby driving pericycle cell dedifferentiation—a key step in LR initiation.^[^ ^47^, ^57^, ^58^ ^]^ This hypothesis aligns with the observed auxin‐independent LR promotion by PNC (Figure 5A,C), suggesting a novel regulatory axis that bypasses traditional hormonal control.

### 3.3 Auxin Distribution is Not Changed in the Background of Nanoceria‐Promoted LR Formation

Auxin is an important regulator of LR development, orchestrating the initiation and emergence of LRP through precise spatiotemporal gradients. It activates pericycle cell division via auxin influx/efflux carriers (PINs and AUX/LAXs), induces cell wall remodeling enzymes, and coordinates cell fate transitions through transcriptional regulators like ARF7/19 and IAA14.^[^ ^59^, ^60^ ^]^ Traditional strategies to enhance LR density—such as exogenous auxin application or genetic overexpression of auxin‐responsive genes—often disrupt auxin homeostasis, leading to pleiotropic effects on shoot growth, flowering, or stress responses.^[^ ^36^, ^61^ ^]^ Previous studies have demonstrated that PNC increased salicylic acid (SA) levels in rapeseed seeds, enhancing salt tolerance,^[^ ^62^ ^]^ suggesting that PNC can affect hormone levels in plants. While whether PNC can affect auxin level or its distribution to promote LR formation is still unknown.

Out of our expectations, the results in this study showed that PNC promote LR formation without altering auxin level and its distribution. *DR5pro::GFP* imaging confirmed no significant changes in auxin gradients in PNC‐treated roots (Figure 5A), and PNC failed to rescue LR suppression caused by the auxin transport inhibitor NPA (Figure 5C). This auxin‐independent action is mechanistically distinct from conventional approaches. By targeting redox signaling rather than hormonal pathways, PNC avoid the systemic side effects of auxin manipulation. For example, auxin overaccumulation can inhibit primary root growth or delay flowering,^[^ ^63^ ^]^ whereas PNC enhances LR proliferation without compromising root‐shoot balance (Figure 2A). Overall, these findings demonstrated the potential of nanobiotechnology approach to bypass pleiotropic hormone effects by targeting stress‐responsive pathways, offering a novel strategy for crop improvement rooted in redox biology avoiding possible side effects from hormone applications.

## 4 Conclusion

In this study, we examined the effects of CeO_2_ nanoparticles on ROS and auxin distribution, focusing on their role in promoting lateral root (LR) formation in *Arabidopsis thaliana*. Three types of nanomaterials were tested: poly(acrylic) acid coated cerium oxide nanoparticles (PNC, 6.5 ± 1.7 nm, −41.5 ± 3.0 mV), aminated PNC (ANC, 6.9 ± 0.6 nm, 28.3 ± 0.6 mV), and bulk cerium oxide nanoparticles (BNC, 84.9 ± 1.9 nm, −5.5 ± 0.3 mV). Only PNC significantly promoted LR formation, which was associated with modulation of ROS, particularly O₂^•−^ in LRP, and was independent of auxin signaling. Transcriptomic analysis indicated that PNC treatment enhances LR development by regulating genes involved in ROS homeostasis. These findings suggest that nanoceria‐based approaches to stimulate LR formation differ from conventional methods and highlight the potential of PNC in enhancing root growth for agricultural applications.

## 5 Experimental Section

### Preparation of PNC, ANC, and BNC

The poly (acrylic acid) nanoceria (PNC) were synthesized employing a previously described methodology by Wu et al. with slight modifications.^[^ ^27^ ^]^ In brief, 1.08 g cerium nitrate (Sigma–Aldrich, 99%) was dissolved in 2.5 mL of deionized water (solution A); 4.5 g of polyacrylic acid (1800 MW, Sigma–Aldrich) was dissolved in 5.0 mL of deionized water (solution B). Mix the two solutions A and B well at 2000 rpm for 15 min using a vortexer, and add the mixture dropwise into a 50 mL beaker containing 15 ml of 30% ammonium hydroxide solution. Stir the mixture at room temperature at 500 rpm for 24 h. Divide it into 1.5 mL centrifuge tubes and centrifuge at 4500 r for 1 h. Discard ≈100 µL of liquid at the bottom of the centrifuge tube. Aspirate the supernatant into an ultrafiltration tube (MWCO 30K, Millipore Inc.), then centrifuge it at 4500 rpm for 6 cycles (one cycle every 45 min). After the cycle is over, collect the supernatant which is the PNC solution. Measure the absorption peak of the collected solution with a spectrophotometer (UV‐1800, AOE). Then, calculate PNC concentration according to the Beer‐Lambert law (see below for details). Finally, store it in a refrigerator at 4 °C.

The synthesis of amino nanoceria (ANC) followed the methods outlined by Asati et al. with some modifications.^[^ ^28^ ^]^ Simply, Mix 3.5 mL of 5 mM PNC with 1.5 mL of ultrapure water at 500 rpm for 2 min at room temperature. Then, 306.8 mg of 1‐ethyl‐3‐(3‐dimethylaminopropyl)carbodiimide (Sigma–Aldrich) (EDC) was dissolved in 0.5 mL of MES buffer (100 mm, pH 6.0), which was added dropwise to the mixture and stirred for 4 min. Then, 800 mm (6 mL) ethylenediamine (EDA, 99%, Sigma–Aldrich) (adjusted to pH 6.8 with HCl) was added dropwise to the final reaction mixture under constant stirring at 500 rpm, and stirring was continued for 3 h. Transfer the resulting solution to a 1.5 mL centrifuge tube and centrifuge at 4500 rpm for 15 min to remove any debris and large clumps. Supernatants were purified by removing excess EDA and other reagents by centrifugation at 4,500 rpm (Allegra X30, Beckman) for 5 cycles (15 min per each cycle) using ultrafiltration tubes (MWCO 30K, Millipore Inc.). After the cycle was over, the absorption peak of the solution was measured with a spectrophotometer, the concentration was calculated according to the Beer‐Lambert law, and finally stored in a refrigerator at 4 °C for future use. Bulk nanoceria (BNC) were commercial cerium oxide nanoparticles purchased from the company (Sigma–Aldrich). The marked particle size was <25 nm, and the measured particle size was about 60–70 nm, with a weak negative charge.

### Preparation of DiI‐PNC

Label PNC with DiI (1,1′‐dioctadecyl‐3,3,3′,3′tetramethylindocarbocyanine, 1,1′‐dioctacyl‐3,3,3′,3′tetramethylindocarbocyanine, a fluorescent protocol) according to previously published methods.^[^ ^27^ ^]^ Specifically, put 4 mL, 0.5 mm PNC and 200 µL, 0.3 mg mL^−1^ DiI (dissolved in DMSO) into a 20 mL glass vial and mix at 1000 rpm for 1 min. Purify the resulting mixture using a 10 kDa filter (4500 rpm, every 5 min, at least five times) to remove free chemicals. Finally, the marked DiI‐PNC was obtained, and the characteristic peak of DiI‐PNC was detected with a UV spectrophotometer to determine the successful labeling of DiI.

### Characterization of PNC, ANC, BNC, and DiI‐PNC

The characterization methods of PNC, ANC, BNC, and DiI‐PNC were based on previous methods with some adjustments in details.^[^ ^27^ ^]^ The absorbance of the final PNC, ANC, and DiI‐PNC solutions was measured with an ultraviolet‐visible spectrophotometer (UV‐1800, AOE). The concentration of PNC, ANC, and DiI‐PNC was analyzed via the Beer‐Lambert law (A = εCL). Here, A represents the absorbance at 271 nm (for PNC and DiI‐PNC) and 264 nm (for ANC), ε denotes the molar absorption coefficient of PNC (3 cm^−1^ mM^−1^), L signifies the optical path length (cuvette width, 1 cm), and C stands for the molar concentration of the measured PNC, ANC, and DiI‐PNC. The hydrodynamic diameter (DLS size) and zeta potential were evaluated using a 90 Plus PALS instrument (Brookhaven Instruments Corporation, USA). For TEM imaging, 20 µL of PNC, ANC, and BNC were applied onto a holey carbon‐coated copper grid, followed by imaging using a FEI Talos microscope operating at 300 kV. PNC and ANC were freeze‐dried into powders. X‐ray photoelectron spectroscopy (XPS) characterization were conducted (Scientific Compass Company, Hangzhou, China), and the obtained XPS data were analyzed using Avantage software (version 6.8.1.4) with peak deconvolution. Following the method described by Korsvik et al. (2007), the valence states of cerium elements were assigned based on the NIST XPS database. The X‐ray diffraction (XRD) patterns of the used CeO₂ nanoparticles were recorded using a Bruker D8 Advance X‐ray Diffractometer with Cu Kα radiation (λ = 1.540 Å). Measurements were performed in the 2θ range from 20° to 80°, operating at 40.0 kV and 30.0 mA. Using a Nicolet iS50 FTIR spectrometer, Fourier transform infrared (FT‐IR) spectra of the used CeO₂ nanoparticles were conducted with scanning wavenumber range of 450 to 4000 cm⁻¹.

### Cultivation and treatment of *Arabidopsis*, Rapeseed, and Rice

The *Arabidopsis thaliana* in this study was of type Col‐0. *Arabidopsis* seeds were soaked in 70% ethanol for 10 s, washed twice in sterile water, soaked in 5% NaClO for 10 min, and washed three times in sterile water. The seeds were evenly sprinkled into the MS square medium, dried on the surface, vernalized at 4 °C in a dark environment for 3 days, and then placed in the light (23 °C, 14/10 h, 200 µmol m^−2^ s^−1^) for 5 days.^[^ ^29^ ^]^ After the *Arabidopsis* seedlings grew in growth chamber (full spectrum 395−800 nm; Boante company, Wuhan, China) for 5 days, *Arabidopsis* seedlings with uniform growth status and approximately the same root length were moved to the experimental treatments [PNC, ANC, BNC, CeCl_3_, DiI‐PNC, DiI‐ANC, H_2_O_2_, DPI, NAA (naphthaleneacetic acid), and NPA (N‐1‐naphthylphthalamic acid)] and the control treatment (adding corresponding volume of solvent) MS solid medium. 6 seedlings per plate, and 4 plates per treatment. After 7 days, take images of *Arabidopsis* seedlings with a camera. The root length and number of *Arabidopsis thaliana* in each treatment were counted by Image J software. The dry and fresh weight were recorded accordingly.

The specific method of PNC treated rapeseed and rice seedlings: After germinating in the germination box for 3 days, select the seedlings with uniform growth for hydroponic cultivation. Then, set up the PNC treatment and the control treatment respectively. After 6 days’ treatment, take out the seedlings to record the root morphology.

### Imaging of ROS in *Arabidopsis* Roots

Imaging of ROS in *Arabidopsis* roots using confocal laser microscopy according to previously published methods with some modifications.^[^ ^27^ ^]^ Dihydroethidium (DHE) and 2iumnfdichlorodihydrofluorescein diacetate (H_2_DCFDA) were used as fluorescent dyes for O₂^•−^ and H_2_O_2_, respectively. To spatially resolve ROS fluorescence signals in root tissues, roots were stained with lipophilic membrane dye FM 4–64 (N‐(3‐triethylammoniumpropyl)‐4‐(6‐(4‐(diethylamino) phenyl) hexatrienyl) pyridinium dibromide; Thermo Fisher). *Arabidopsis* roots grown on MS, PNC (0.1 mm) ± MS medium for 7 days were soaked in 25 µm H_2_DCFDA or 10 µm DHE dye (diluted with 10 mm TES, pH = 7.5), and incubated for 30 min in the dark. After the initial 30 min incubation period, roots were transferred to tubes containing 3 µm FM 4–64 in aqueous solution and stained for 1 h at 25 °C in darkness. After the incubation, rinse with TES three times, put it into a slide (a drop of perfluoronaphthylamine (PFD) was added to the slide in advance to enhance the effect of fluorescence imaging), cover with a cover slip, and make sure that there is no bubble. The laser confocal microscope was set as follows: 40x objective lens, 488 nm excitation light; emission channel for DHE: 520–580 nm; emission channel for DCF: 500–550 nm, and emission channel for FM 4–64: 610–630 nm. 4–6 repetitions were made, and the fluorescence intensity of DCF and DHE was calculated using Image J software.

### RNA‐Seq and Data Analysis

Total RNA from plant samples was extracted using the RNAprep Pure Plant Kit (Tiangen, Beijing, China), and RNA samples were subsequently sent to BioMarker (BMK, Beijing) for sequencing, with three biological replicates. Gene functional annotations were based on the following databases: Nr (NCBI non‐redundant protein sequences), Pfam (Protein family), KOG/COG (Clusters of Orthologous Groups of proteins), Swiss‐Prot (manually annotated and reviewed protein sequences), KO (KEGG Ortholog database), and GO (Gene Ontology). The fragments per kilobase of transcript per million mapped reads (FPKM) were calculated using the formula:

(1) $$FPKM=\frac{\mathrm{cDNA}\;\mathrm{fragments}}{\mathrm{Mapped}\;\mathrm{fragments}\left(\mathrm{Millions}\right)\times \;\mathrm{Transcript}\;\mathrm{length}\left(\mathrm{kb}\right)\;}$$

Differential expression analysis between two conditions/groups was performed using DESeq2, which employs a negative binomial distribution model to identify differentially expressed genes from digital gene expression data. P‐values were adjusted for multiple testing using the Benjamini‐Hochberg method to control the false discovery rate. Genes with an adjusted *p*‐value < 0.01 and a fold change ≥ 2 were considered differentially expressed. Gene Ontology (GO) enrichment analysis of differentially expressed genes (DEGs) was conducted using the clusterProfiler package, which implements the Wallenius non‐central hypergeometric distribution to account for gene length bias.^[^ ^30^ ^]^

### DR5pro::GFP *Arabidopsis* GFP Fluorescence Monitoring

The 5‐day‐old DR5pro::GFP transgenic *Arabidopsis seedlings* were moved to the MS solid medium containing PNC, and the GFP fluorescence in the root system was monitored at 24, 36, and 48 h after culture, respectively. Fluorescence monitoring was performed using a Leica laser confocal SP8 with excitation at 514 nm and emission at 550–615 nm.^[^ ^31^ ^]^

### Statistical Analysis

All data were represented as mean ± SE (n = biological replicates) and analyzed using Excel 2019 and SPSS 23.0. The comparison was performed by an independent sample t‐test (two‐tailed) or one‐way ANOVA based on Duncan's multiple range test (two‐tailed). * and ** represent *p* < 0.05 and *p* < 0.01, respectively. Different lowercase letters mean the significance at *p* < 0.05.

## Conflict of Interest

The authors declare no conflict of interest.

## Author Contributions

G.L. contributed to the conceptualization, data curation, formal analysis, investigation, methodology, validation, visualization, writing – original draft, and writing – review and editing. J.Q. was involved in investigation and validation. W.X. contributed to investigation and data curation. L.C. provided resources. A.N., Z.X., J.G., and Z.L. contributed to writing – review and editing. H.W. contributed to supervision, conceptualization, funding acquisition, and writing – review and editing. All authors edited the manuscript and approved the final version.

## Supporting information



Supporting Information

## Data Availability

The data that support the findings of this study are available in the supplementary material of this article.
