# Supplementary material for: Negatively but Not Positively Charged Nanoceria Promoted Lateral Root Growth via Modulating the Distribution of Reactive Oxygen Species Rather than Auxin
Source: Glob Chall. 2025 Jul 20;9(9):e00186. doi: 10.1002/gch2.202500186 (PMC12418346; doi:10.1002/gch2.202500186)
Supplement: Supplementary file 1 — Supporting Information [file GCH2-9-e00186-s001.docx]

Supporting Information for

**Negatively but not positively charged nanoceria promoted lateral root growth via modulating the distribution of ROS rather than auxin**

Guangjing Li^1,2^, Jie Qi^1,2^, Wenying Xu^1,2^, Linlin Chen^1,2^, Ashadu Nyande^1,2^, Zhouli Xie^1,2^, Jiangjiang Gu^2,3^, Zhaohu Li^1,2^, Honghong Wu^1,2,4,5^*

^1^ National Key Laboratory for Germplasm Innovation & Utilization of Horticultural Crops, The Center of Crop Nanobiotechnology, College of Plant Science & Technology, Huazhong Agricultural University, Wuhan, China, 430070

^2^ Hubei Hongshan Laboratory, Wuhan, China, 430070

^3^ College of Chemistry, Huazhong Agricultural University, Wuhan, China, 430070

^4^ Shenzhen Institute of Nutrition and Health, Huazhong Agricultural University, Shenzhen, China, 511464

^5^ Shenzhen Branch, Guangdong Laboratory for Lingnan Modern Agriculture, Genome Analysis Laboratory of the Ministry of Agriculture, Agricultural Genomics Institute at Shenzhen, Chinese Academy of Agricultural Sciences, Shenzhen, China, 511464

* Corresponding author: honghong.wu@mail.hzau.edu.cn

**Figure S1**


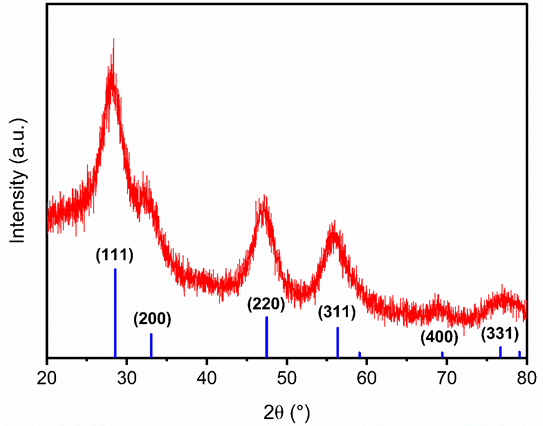
**Figure S1.** XRD pattern of PNC.

**Figure S2**


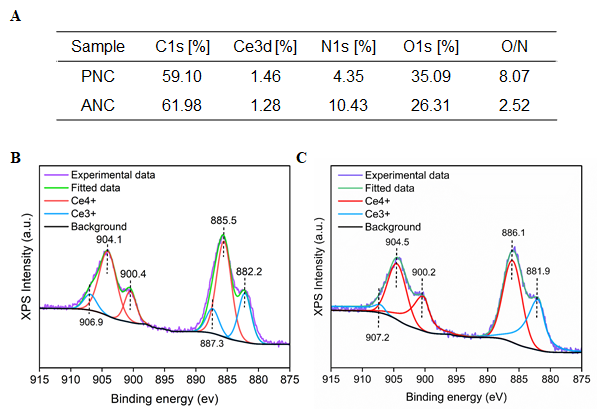


**Figure S2.** XPS analysis of PNC and ANC. A, Surface composition from XPS spectra (in atomic percent) of PNC and ANC. B-C, High-resolution Ce XPS spectrum of PNC (B) and ANC (C).

**Figure S3**


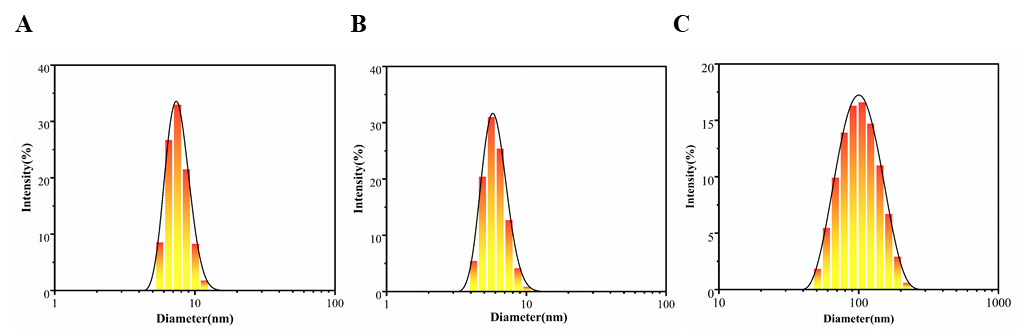


**Figure S3.** DLS particle size distribution of PNC, ANC, and BNC. Hydrodynamic diameter of (A) PNC, (B) ANC, and (C) BNC. PNC and ANC were filtered through a 20 nm filter. At least three batches were tested. Mean ± SE (n = 3).

**Figure S4**


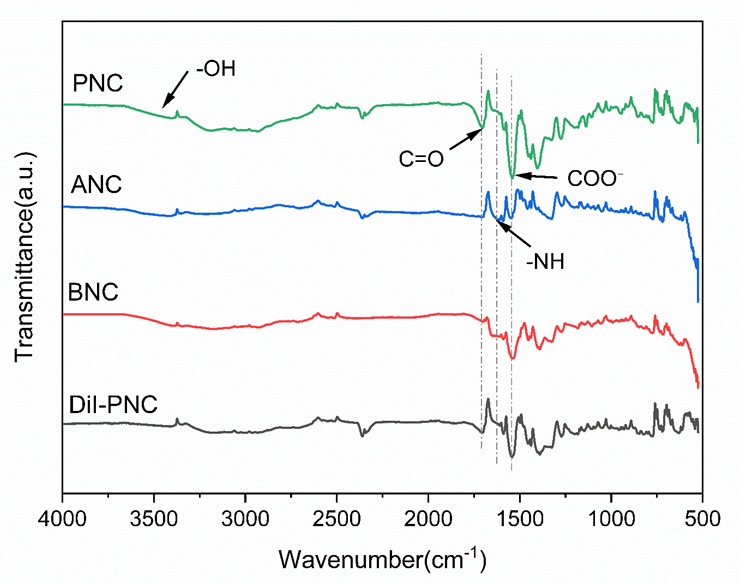
**Figure S4**. FTIR characterization of PNC, ANC, BNC and DiI-PNC.

**Figure S5**


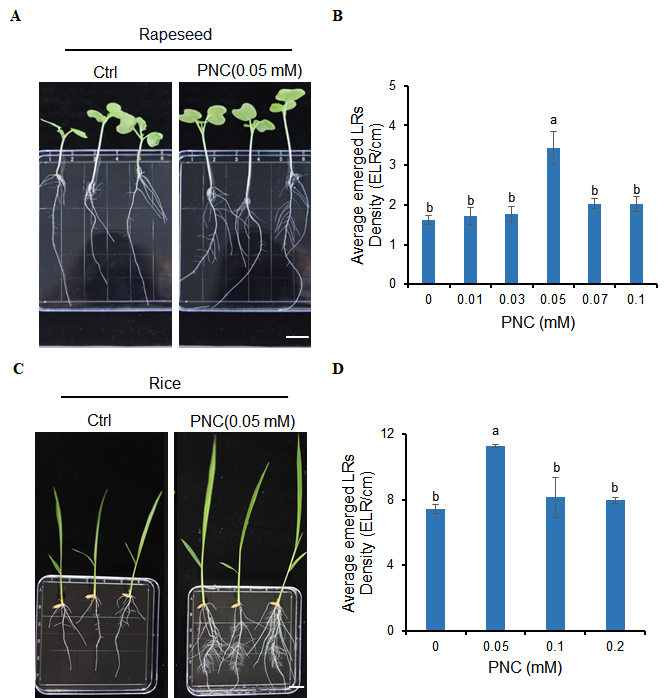


**Figure S5**. PNC promote root growth of rapeseed and rice. A and C. The phenotype of rapeseed (A) and rice (C) treated with PNC for 6 days. Scale bar, 1.5 cm. B and D. Emerged LRs density of rapeseed (B) and rice (D) treated with PNC for 6 days. Mean ± SE (n = 6 biological replicates. Each replicate contains one plant). The comparison was performed by one-way ANOVA based on Duncan's multiple range test. Different lowercase letters mean the significance at *p* < 0.05.

**Figure S6**


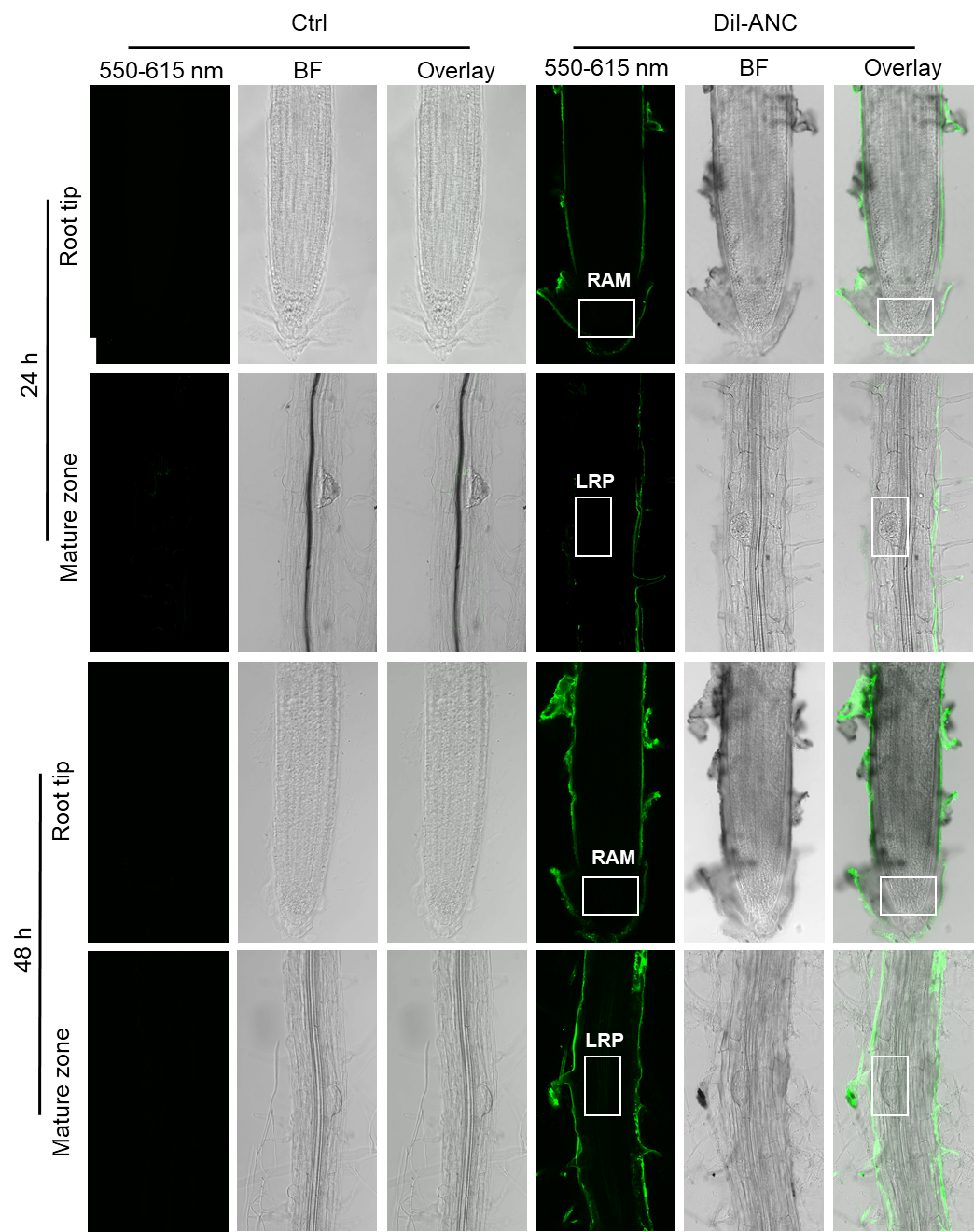
**Figure S6.** The distribution of ANC in *Arabidopsis* roots. Representative confocal images showing the distribution of ANC in Arabidopsis roots at 24 h and 48 h post-treatment. White box indicated the RAM and LRP, respectively. Scale bar = 50 μm. Mean ± SE (n = 6 biological replicates. Each replicate contains plant).

**Figure S7**


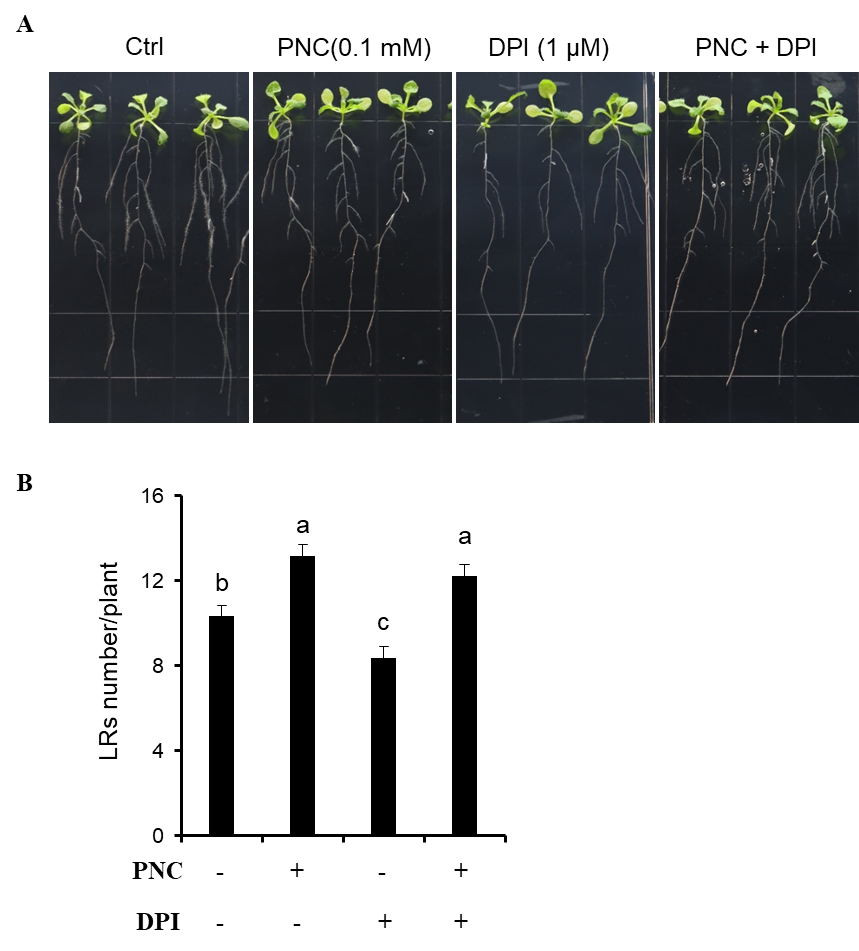


**Figure S7.** PNC can relieve the inhibitory effect of DPI on lateral root formation in Arabidopsis. A, Phenotypes of Arabidopsis seedlings treated with active substance (PNC or DPI) for 7 days. B, The number of lateral roots in Arabidopsis for 7 days. Scale bar, 1 cm. Mean ± SE (n = 6 biological replicates, each replicates contains three plants). The comparison was performed by one-way ANOVA based on Duncan's multiple range test. Different lowercase letters mean the significance at *p* < 0.05.
